# The microbial metabolite trimethylamine N-oxide and the kidney diseases

**DOI:** 10.3389/fcimb.2025.1488264

**Published:** 2025-03-11

**Authors:** Jin-Qi Su, Xiang-Qi Wu, Qi Wang, Bo-Yang Xie, Cui-Yan Xiao, Hong-Yong Su, Ji-Xin Tang, Cui-Wei Yao

**Affiliations:** ^1^ Guangdong Provincial Key Laboratory of Autophagy and Major Chronic Non-communicable Diseases, Institute of Nephrology, Affiliated Hospital of Guangdong Medical University, Zhanjiang, Guangdong, China; ^2^ Key Laboratory of Prevention and Management of Chronic Kidney Diseases of Zhanjiang City, Institute of Nephrology, Affiliated Hospital of Guangdong Medical University, Zhanjiang, Guangdong, China

**Keywords:** gut microbiome, TMAO, kidney injury, chronic kidney disease, end-stage renal disease, inflammatory mechanisms

## Abstract

Trimethylamine N-oxide (TMAO), a metabolite, is a co-metabolite produced by both gut microbiota and livers, originating from foods rich in choline or carnitine. Emerging evidence suggests that TMAO may play a role in the pathogenesis of various kidney diseases, including acute kidney injury and chronic kidney disease. Research has demonstrated that heightened levels of TMAO are correlated with a heightened likelihood of kidney disease advancement and cardiovascular incidents among individuals with chronic kidney disease. Furthermore, TMAO has been observed to stimulate inflammation, oxidative stress, and fibrosis in animal models of kidney disease. Mechanistically, TMAO may contribute to kidney disease pathogenesis by inhibiting autophagy, activating the NLRP3 inflammasome, and inducing mitochondrial dysfunction. Therefore, targeting TMAO may represent a promising therapeutic strategy for the treatment of kidney diseases. Future studies are needed to further investigate the role of TMAO in kidney disease pathogenesis and to develop TMAO-targeted therapies for the prevention and treatment of kidney diseases.

## 1 Introduction

**Figure 1:**
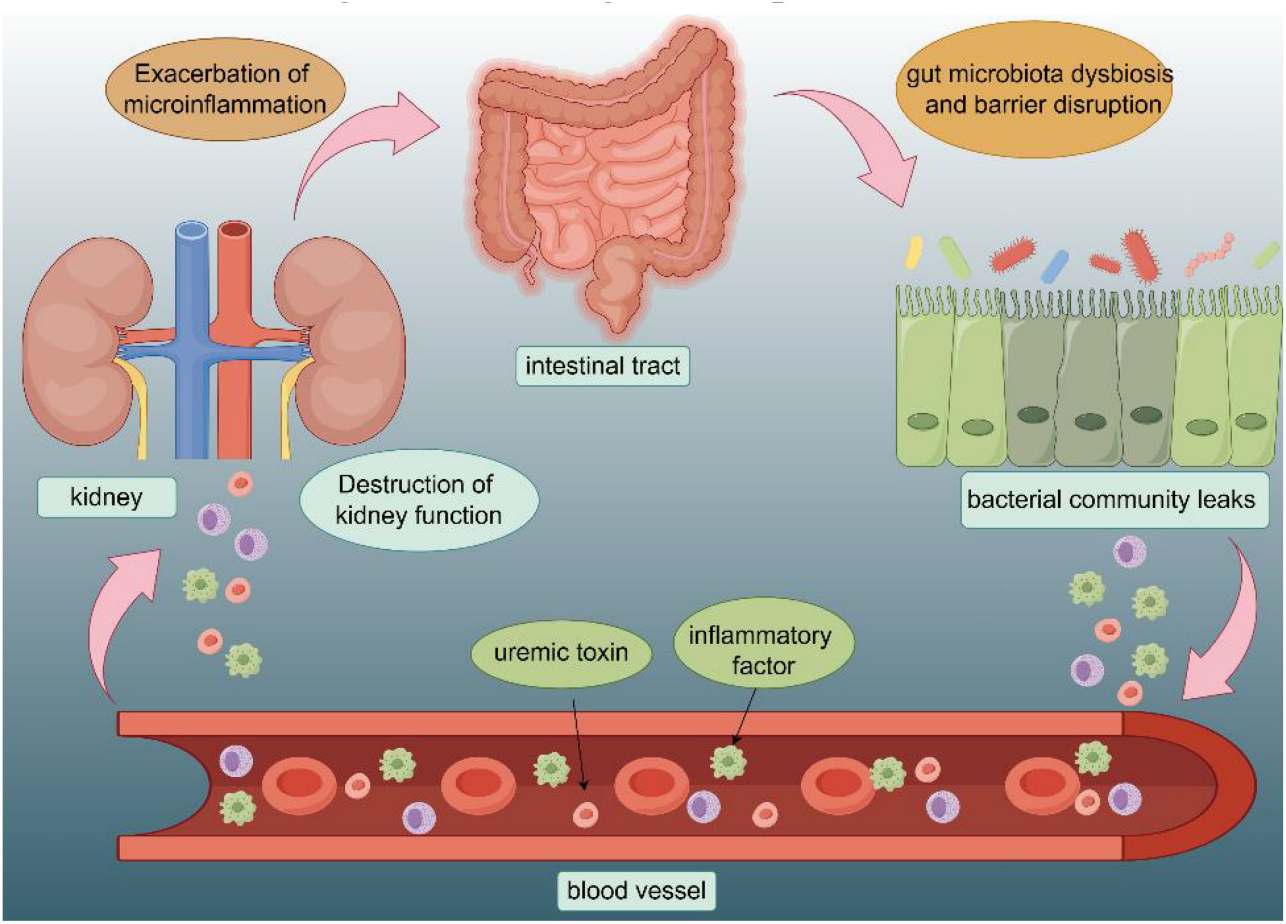
Interaction of intestinal structures with the renal system: The intestinal mucosa is where the gut microbiota is most active, and is often affected by diet, toxins, medications, and other factors. Gut hormones and metabolites affect the kidneys through the bloodstream. The disruption of kidney function will in turn exacerbate the microinflammatory state of the gut as well as disturbances in the gut microflora.

Kidney disease poses a substantial public health concern, as approximately 10% of the worldwide population is afflicted by chronic kidney disease (CKD) (Rosner et al., 2021). The pathogenesis of kidney diseases is complex, involving interactions between genetic, environmental, and lifestyle factors. Gut microbiota is an important regulator of the human immune system, consequently, the concept of the kidney-gut axis has been proposed and is gradually gaining attention (**Figure 1**) (Anand and Mande, 2022). During the course of patients with kidney disease, due to the microinflammatory state caused by their immune mechanisms, they are more likely to have abnormal distribution of intestinal bacteria and intestinal microecological disorders, which are called gut microbiota dysbiosis (Belvoncikova et al., 2022). This state can exacerbate the microinflammatory state of the patient’s body, thus increasing the production of renal toxins, which affects the patient’s prognosis avid continually recurring (Huang et al., 2022).

Recent research indicates that the gut microbiota and their metabolites may significantly influence the onset and advancement of kidney diseases. Of particular interest is trimethylamine N-oxide (TMAO), a metabolite that has attracted growing attention for its potential role in the progression of kidney diseases. TMAO is a microbial metabolite originating from dietary sources rich in choline and carnitine. Within the gastrointestinal tract, gut bacteria facilitate the conversion of choline and carnitine into trimethylamine (TMA), which is further metabolized in the liver to produce TMAO (Wang et al., 2011). After its formation, TMAO is distributed in the blood and accumulates in diverse tissues, notably the kidney. Extensive research has substantiated a correlation between elevated levels of circulating TMAO and the onset and advancement of kidney ailments such as acute kidney injury (AKI), CKD, and end-stage renal disease (ESRD). Furthermore, clinical investigations have revealed that the high level of TMAO is linked to an augmented likelihood of unfavorable renal outcomes, including deterioration in renal function and mortality in individuals with kidney disorders (Kalantar-Zadeh et al., 2021).

While significant evidence connects TMAO to kidney disease, the exact mechanisms of how it causes kidney injury remain unclear. Experimental studies utilizing animal models have elucidated that TMAO has the potential to induce oxidative stress, inflammation, endothelial dysfunction, and fibrosis in the kidneys, ultimately resulting in compromised renal function (Lau et al., 2018). Furthermore, recent investigations have shown that TMAO may influence intracellular signaling pathways and cellular mechanisms within the kidney, such as autophagy, inflammasome activation, and mitochondrial function, all of which play a role in the development of kidney diseases (Wang et al., 2021). Therefore, understanding the molecular pathways through which TMAO affects renal homeostasis is essential for the development of precise therapeutic strategies.

In conclusion, the gut microbial metabolite TMAO has emerged as a potential contributor to the progression of kidney diseases. Understanding the role of TMAO in kidney diseases may lead to the development of novel therapeutic approaches that target TMAO metabolism and signaling pathways for the prevention and treatment of kidney diseases. Therefore, further investigation into the interactions between TMAO and kidney function may advance our understanding of the role of TMAO in kidney diseases and to develop potential interventions to improve renal function.

## 2 The sources, metabolism, and primary detection methods of TMAO

### 2.1 The source of TMAO

The human body has the capacity to acquire TMAO from diverse origins, with dietary intake being a prominent source of TMAO in the peripheral blood. Foods rich in choline, lecithin, and L-carnitine, such as red meat, eggs, dairy products, and saltwater fish, are metabolized by gut microbes during digestion to produce TMAO in liver (Wang et al., 2011). The breakdown and metabolism of nutrients by gut bacteria via microbial TMAO generation is influenced significantly by an individual’s dietary choices, ultimately impacting the levels of TMAO present in their peripheral blood.

In addition to dietary sources, pharmaceutical products rich in choline may also play a role in the synthesis of TMAO within the body (Hampel et al., 2018). Certain medications or supplements containing high concentrations of choline have the potential to increase TMAO levels. Initially considered an insignificant byproduct of choline metabolism, TMAO has since been linked to hypertension, atherosclerosis, coronary artery disease, diabetes, and renal failure. This highlights the need to study TMAO’s origins and its health impacts (Nowiński and Ufnal, 2018).

### 2.2 Metabolism of TMAO

**Figure 2:**
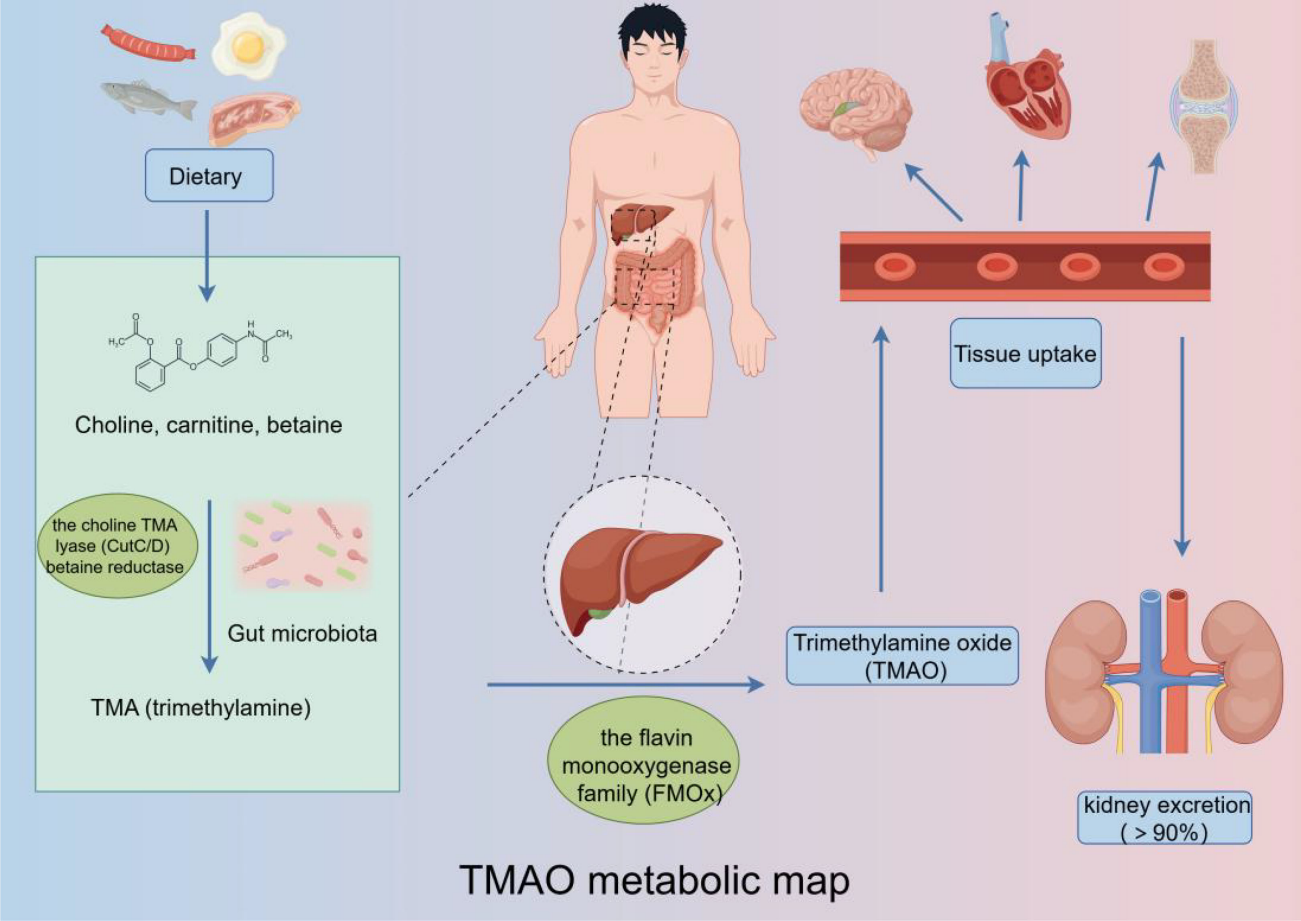
TMAO metabolic pathway: Trimethylamine-containing nutrients are converted to trimethylamine N-oxide by a gut microbiota-dependent initial step, followed by conversion to trimethylamine N-oxide by host liver flavin monooxygenase, which then enters the bloodstream to exert its effects on various systems.

The metabolic pathway of TMAO is an intricate process involving the enzymatic conversion of dietary choline to TMA by specific gut microorganisms (**Figure 2**). These microorganisms harbor enzymes, such as CutC and betaine reductase, which facilitate the breakdown of choline and subsequent release of TMA. Following its formation, TMA is absorbed into the bloodstream through the intestinal epithelium and transported to the liver. Within the hepatic environment, TMA is subjected to oxidation by flavin-containing monooxygenases (FMO), resulting in the conversion of TMA to TMAO, the fully oxidized derivative of the compound (Gatarek and Kaluzna-Czaplinska, 2021; Benson et al., 2023).

One mechanism by which the body maintains TMAO homeostasis is through excretion, the kidney is the prominent organ to eliminate TMAO. While the majority of TMAO is excreted through kidney in the metabolic cycles, a small portion is also eliminated through alternative routes such as sweat, feces, and respiratory metabolism. These additional excretion pathways collectively contribute to maintain TMAO balance in the body (Gessner et al., 2020).

In summary, TMAO metabolism involves converting dietary choline to TMA by gut microbes, oxidizing TMA to TMAO in the liver, and primarily excreting TMAO via the kidney, with minor elimination through sweat, feces, and breath. Understanding this process is essential for researching TMAO’s physiological roles and regulating its levels.

### 2.3 Primary detection methods of TMAO

The detection of TMAO can be achieved through various methods, including enzyme-linked immunosorbent assay (ELISA), Liquid chromatography-mass spectrometry (LC-MS/MS), Nuclear magnetic resonance spectroscopy (NMR), gas chromatography, and electrochemical sensors.

The ELISA is a frequently utilized method in the field of TMAO investigation. This technique relies on the utilization of specialized antibodies that have an affinity for TMAO, enabling its identification and measurement. LC-MS/MS is commonly employed in research investigations owing to its exceptional sensitivity and specificity. NMR serves as a non-invasive analytical tool for detecting TMAO, relying on the detection of distinctive signals emitted by TMAO under the influence of a powerful magnetic field. NMR offers valuable insights into the structural and dynamic properties of TMAO, thereby contributing to a comprehensive understanding of this compound (Du et al., 2017; Rox et al., 2021; Yang et al., 2021; Aksoyalp et al., 2023). Additionally, less common methods for detecting TMAO are gas chromatography, which separates sample components via gas phase partitioning, and electrochemical sensors, which identify changes in electrical properties when TMAO reacts with specific electrodes (Fiori et al., 2018; Çorman et al., 2022).

In conclusion, a variety of techniques can be utilized for the detection of TMAO (Fabresse et al., 2020; Tang et al., 2022). Ongoing enhancements and innovations in these methodologies are anticipated to propel progress in TMAO investigation and its prospective utility in the healthcare sector.

## 3 TMAO and kidney diseases

### 3.1 TMAO and AKI

**Figure 3:**
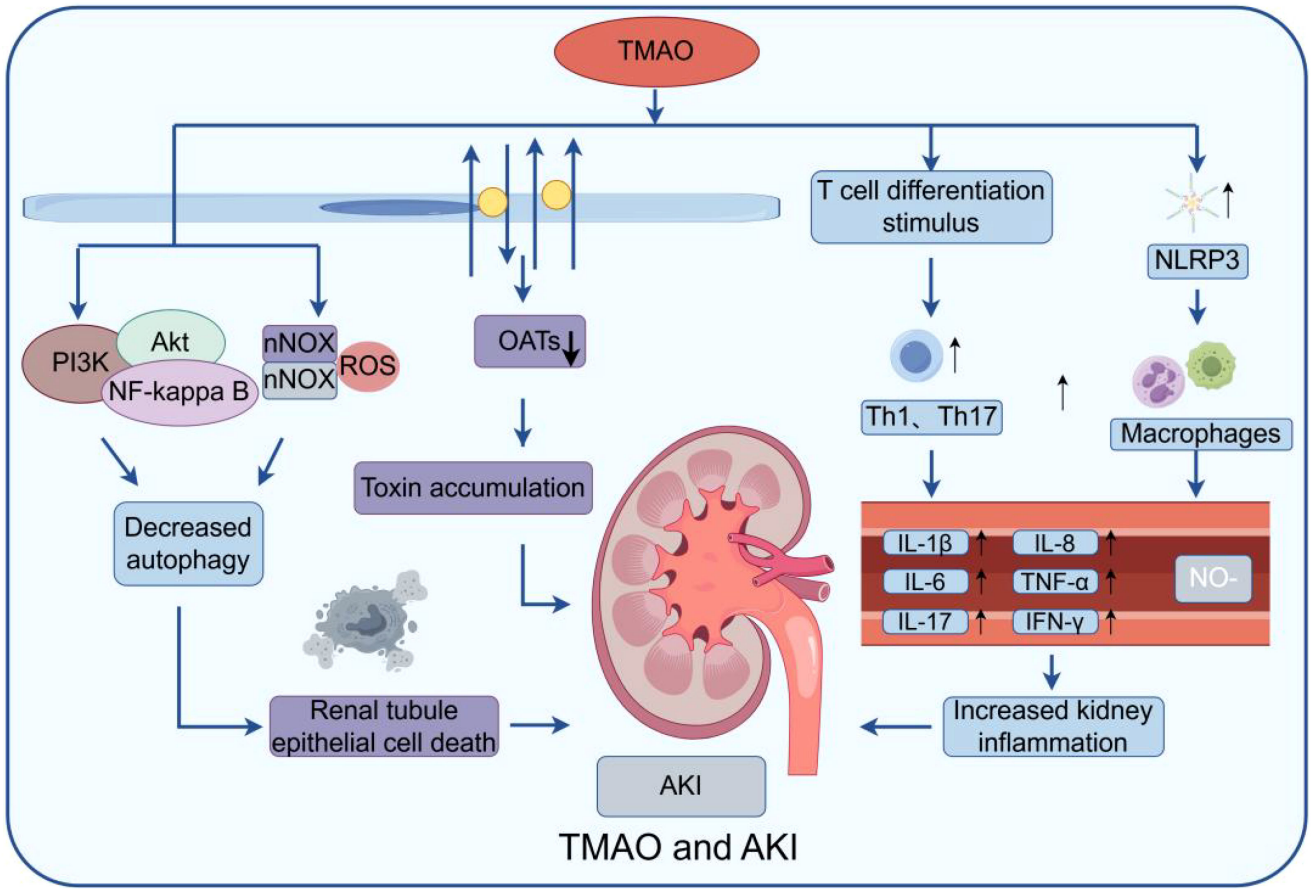
Model of TMAO effect on AKI: The figure mainly shows that TMAO can stimulate NLRP3 in macrophages and stimulate T cell differentiation, produce a variety of inflammatory factors, and aggravate the inflammatory response. At the same time, by inducing the generation of reactive oxygen species (ROS) and activating PI3K/Akt/NF-κB signaling pathway, the imbalance of autophagy process can lead to increased death of renal tubular epithelial cells and damage kidney function. Secondly, it can affect the function of some transporters, such as organic anion transporters (OATs) and organic cationic transporters (OCTs), leading to the accumulation of waste in the cell, further aggravating cell damage and inflammatory reaction.

Acute kidney injury (AKI) is characterized by a rapid decline in renal function, frequently triggered by ischemia, nephrotoxic medications, sepsis, or other insults to the kidneys. Numerous mechanisms cause AKI, including prolonged organ underperfusion, systemic wasting disorders, toxins, infections, and severe forms of primary glomerular disease (Devarajan, 2023). Recent studies have suggested a potential link between TMAO and AKI (Missailidis et al., 2016). Research conducted in animal models has shown an increase in TMAO levels in the blood of AKI animals. Furthermore, elevated TMAO levels have been detected in AKI patients (Liu et al., 2024). The precise mechanism by which TMAO contributes to AKI remains incompletely elucidated, potentially involving multiple pathways (**Figure 3**).

One potential mechanism is through the activation of inflammatory responses. TMAO promotes the production of pro-inflammatory cytokines like IL-6 and TNF-α, leading to kidney inflammation and damage. It also induces apoptosis in renal cells, disrupts autophagy, and increases oxidative stress, contributing to AKI progression (Shi et al., 2022; Lee et al., 2024). An additional potential mechanism involves the interference with the regulation of renal blood flow. Studies have demonstrated that TMAO can hinder endothelial function and decrease the availability of nitric oxide (NO) (Querio et al., 2022). The decreased bioavailability of NO caused by TMAO may induce renal vasoconstriction and ischemia, ultimately resulting inAKI. Moreover, TMAO disrupts the renal tubular transport system by inhibiting organic cation and anion transporters (OCTs and OATs), essential for reabsorbing and secreting various compounds in the kidneys (Samodelov et al., 2020). TMAO up-regulates NOX2, increasing fibronectin, p65, and Snail, which blocks the G2/M cell cycle and heightens kidney inflammation via the NOX/ROS pathway. Additionally, TMAO damages renal cells through the PI3K/Akt/NF-kappa B pathway. Elevated TMAO in mice also shows continuous CCR2 expression from monocytes, indicating CCR2 as a potential target for TMAO (Ren et al., 2024).

In summary, recent studies indicate a possible connection between TMAO and AKI, with elevated TMAO levels observed in AKI patients. TMAO appears to induce renal inflammation, apoptosis, oxidative stress, and disrupt kidney function. However, more research is needed to confirm these findings and understand the mechanisms involved. Future research should explore TMAO-targeted interventions and its potential as an early biomarker for AKI.

### 3.2 TMAO and CKD

Given the established link between TMAO and AKI, it is crucial to explore the mechanisms by which TMAO may exacerbate CKD progression. CKD is a chronic progressive disease. It is defined by KDIGO as a persistent elevation of urinary albumin excretion (UAE) (30 mg/g, or 3 mg/mmol) or a persistent decrease in glomerular filtration rate (GFR) (less than 60 ml/min, or both) that lasts for more than three months (KDIGO 2022 clinical practice guideline for diabetes management in chronic kidney disease, 2022). In addition, in global health statistics, CKD has gradually become an important cause of death worldwide, affecting 15-20% of adults worldwide, and is often associated with diabetes, hypertension, cardiovascular disease and other chronic diseases (Jankowski et al., 2021; Matsushita et al., 2022). The bidirectional relationship between TMAO and CKD is evidenced by the influence of CKD on TMAO levels, as well as the potential for elevated TMAO levels to contribute to the progression of CKD (Xu et al., 2017; Jia et al., 2019; Pelletier et al., 2019; Lim et al., 2021). Studies show that CKD patients have significantly higher TMAO levels than healthy individuals, which are linked to increased risk of cardiovascular events and mortality (Zeng et al., 2021; Andrikopoulos et al., 2023; Wang et al., 2024). Interestingly, this elevated risk also varies by ethnicity, with a cohort study that CKD patients with high plasma levels of TMAO had a higher mortality risk, and that risk was higher among white people than blacks people (Tang et al., 2015; Dai et al., 2022; Kalagi et al., 2023; Li et al., 2023).

**Figure 4:**
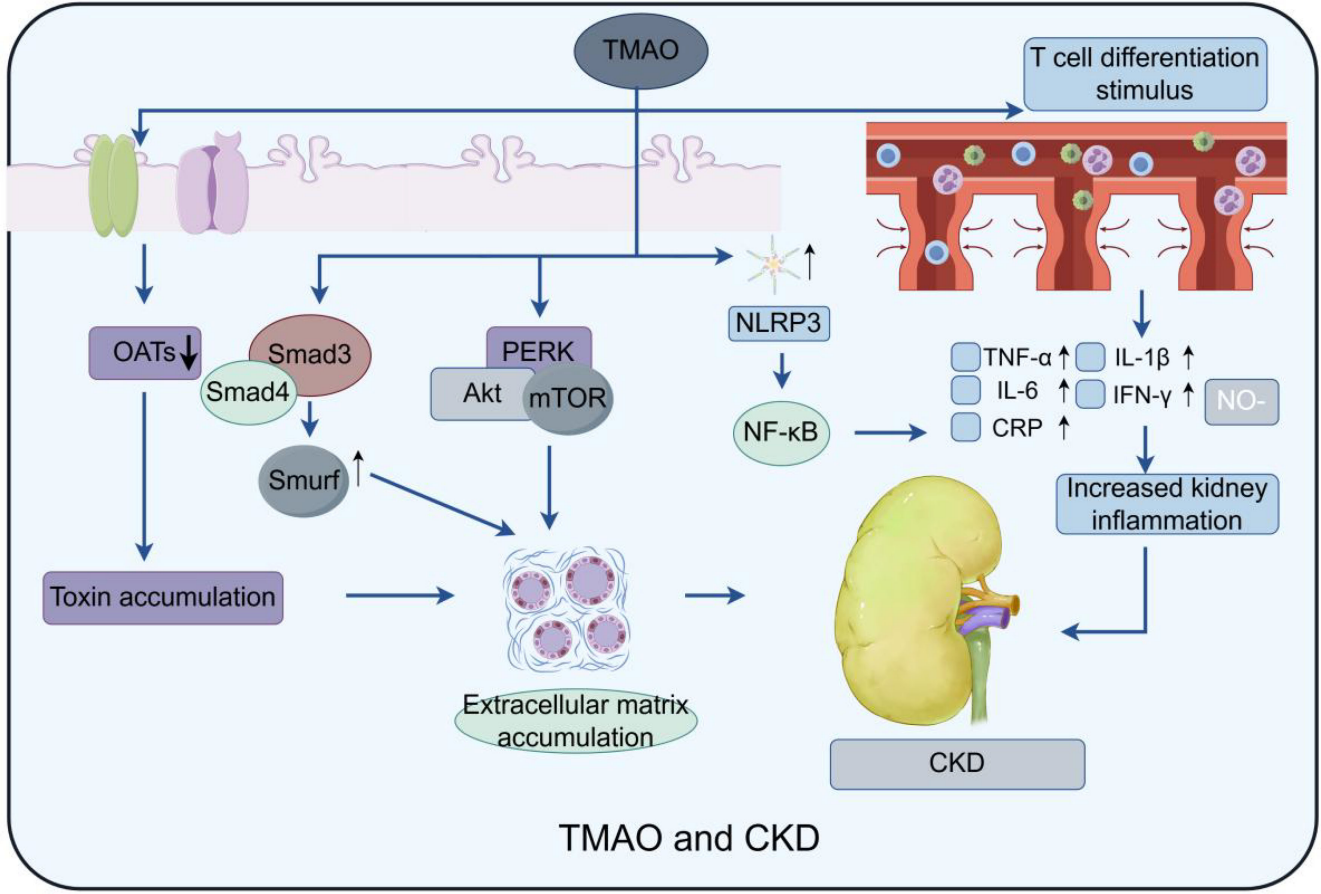
Model of TMAO effect on CKD: Three main pathways are depicted: TMAO activates macrophages by stimulating NLRP3 (NOD-like receptor protein 3) and stimulates T cell differentiation to produce multiple inflammatory factors, such as TNF-α, IL-6, etc., which exacerbate the inflammatory response; At the same time, it not only promotes the formation of NLRP3 inflammasome, but also activates the expression of NF-κB signaling pathway, Caspase-1 activation, PERK/Akt/mTOR pathway, IL-1β and other inflammatory and fibrosis-related factors, thus significantly enhancing the process of renal fibrosis. Finally, the function of the intrarenal transporters is affected, resulting in the accumulation of waste products in the kidney, which may further exacerbate kidney injury and fibrosis.

TMAO may impact CKD development and progression through various mechanisms (**Figure 4**). First, TMAO has been demonstrated to stimulate inflammation and oxidative stress, both of which are significant factors in the progression of CKD (Lau et al., 2018). High TMAO levels can elevate inflammatory factors like TNF-α, MCP-1, IL-1β, IL-6, and IL-18 by activating p38 phosphorylation and upregulating human antigen R (HuR). It is well known that p38 is a core component of the MAPK pathway, and phosphorylated p38 can turn on the transcription of many target genes, such as TNF-α, MCP1, and NLRP3 inflammasome. Cytoplasmic localization of HuR is also controlled by the p38/MAPK pathway. HuR is an RNA-binding protein that binds to the AU-rich element (ARE) of the 3’ untranslated region of mRNA. It can maintain the stability of TNF-α, IL-6, IL-18 and other inflammatory cytokines mRNA. TTP is another RNA binding protein. However, TTP can promote the degradation of these cytokines, while HuR can competitively inhibit TTP (Lai et al., 2022). Second, it was shown that TMAO induces endothelial dysfunction by inhibiting nitric oxide production, increasing reactive oxygen species, and promoting smooth muscle cell proliferation (Li et al., 2022). And TMAO could exacerbate oxidative stress through upregulation of NOX4 and downregulation of SOD. According to studies, TMAO may be elevated HuR to bind to the NOX4 promoter (Shi et al., 2020). Third, TMAO may potentially disrupt the equilibrium of renal electrolytes and water transport by inhibiting sodium and water reabsorption in the kidneys, resulting in sodium retention and volume expansion, characteristic features of chronic kidney disease (Fang et al., 2023).

Besides, TMAO may directly harms kidney structure, causing glomerular hypertrophy, tubulointerstitial fibrosis, and renal inflammation in animals, which promoting the progression of CKD. For instance, TMAO triggers NLRP3 inflammasome formation and activates NF-κB signaling, Caspase-1, the PERK/Akt/mTOR pathway, and IL-1β, leading to increased renal fibrosis (El-Deeb et al., 2019; Zhang et al., 2020; Kapetanaki et al., 2021; Xie et al., 2022). PERK knockdown consistently mitigated TMAO-induced autophagy, apoptosis, and oxidative stress in renal cells. Another study found that high TMAO levels significantly elevated Smad3 in mice, leading to renal fibrosis (Zhang et al., 2021). Further investigation showed that TMAO also increased Smad4, which promotes fibrosis by inducing Smad3-regulated microRNAs and Smad ubiquitination regulatory factor (Chen et al., 2018). TMAO also influences renal cell autophagy and cell cycle regulation. For instance, an *in vitro* study found that TMAO down-regulates Gadd45a expression by mediating the nuclear translocation of Y-box binding protein-1, thus inhibiting cell cycle progression (Wang et al., 2021). In animal studies on hyperuricemic nephropathy, TMAO accelerates renal fibrosis by activating PI3K/AKT/mTOR pathway. Chlorogenic acid can counteract this effect (Dong et al., 2022; Zhou et al., 2022). Current studies have shown that mTOR is comprised of mTOR complex 1 (mTORC1) and mTOR complex 2 (mTORC2). mTORC1 accomplishes metabolic reprogramming by enhancing the biosynthesis of glycolysis, proteins, lipids, and nucleic acids through the activation of S6 kinase. mTORC2 is located upstream of AKT kinase and regulates cell growth and migration by phosphorylating the S473 site of AKT and modulating cytoskeletal proteins. However, the specific mTOR complex that TMAO affects is still under investigation.

In summary, TMAO contributes to CKD progression, indicating that targeting TMAO could be a promising treatment strategy. Reducing TMAO levels may mitigate inflammation, oxidative stress, endothelial dysfunction, and renal abnormalities, and improving patient outcomes. However, more research is necessary to understand the mechanisms and develop effective interventions for regulating TMAO in the blood of CKD patients.

### 3.3 TMAO and ESRD

Building on the understanding of TMAO’s role in CKD, we now delve deeper into its implications for ESRD, highlighting the potential pathways through which TMAO contributes to the complex health challenges faced by ESRD patients. ESRD refers to the end stage of chronic kidney disease caused by various causes. It usually presents with pathological changes of glomerular filtration rate below 15 ml/(min·1.73m^2), accompanied by retention of metabolites and toxins, and disturbance of water, electrolyte and acid-base balance as the main clinical features (Wouk, 2021). ESRD often coexists with comorbidities like cardiovascular disease, anemia, and bone disorders, significantly affecting patient quality of life and survival. TMAO, a biomarker of ESRD, is a co-metabolite produced by both microbiota and livers, originating from choline- or carnitine-rich foods and entering the bloodstream. While healthy kidney can remove TMAO efficiently, the loss of kidney function in ESRD patients leads to its accumulation in blood (Zhang et al., 2020; Zheng et al., 2020; Li et al., 2023).

The elevated levels of TMAO in the blood of patients with ESRD have profound implications for their health status and comorbidities. Specifically, heightened TMAO levels have been linked to a heightened risk of cardiovascular events, including myocardial infarctions and cerebrovascular accidents (Stubbs et al., 2016; Rodrigues et al., 2021). At high concentrations of TMAO, microvascular tight junction proteins in subcutaneous adipose tissue are significantly reduced in ESRD patients, disrupting the intestinal barrier and blood-brain barrier, ultimately leading to clinical manifestations such as depression and cognitive decline. In addition, TMAO may accelerate the process of atherosclerosis and increase the risk of cardiovascular diseases through mechanisms such as promoting inflammation, increasing the expression of scavenger receptors, inhibiting reverse cholesterol transport, and enhancing vascular calcification (Chen et al., 2017; Dicks, 2024; Luo et al., 2024). For instance, Hemodialysis (HD) patients had significantly higher serum TMAO levels than healthy individuals, and HD patients with high abdominal aortic calcification (AAC) scores had notably higher TMAO levels than those with low AAC scores (He et al., 2022). In a subsequent study, PY et al. discovered that elevated serum TMAO levels in HD patients raise carotid-femoral pulse wave conduction velocity (cfPWV), thereby increasing aortic stiffness and the risk of cardiovascular disease (Shafi et al., 2017; Tomlinson and Wheeler, 2017; Huang et al., 2023). Vascular calcification also leads to HD failure and poor prognosis in ESRD patients.

Moreover, studies have demonstrated that TMAO plays a role in promoting inflammatory responses and oxidative stress in the body, which are critical factors in the advancement of kidney disease. TMAO may accelerate renal function decline and increase the risk of complications like anemia, bone disorders, and mortality in ESRD patients. Such as, the elevation of TMAO appears to be associated with the occurrence of PD-associated peritonitis in patients treated with peritoneal dialysis (PD) (Chang et al., 2022). The pathogenesis of increased TMAO concentration on peritonitis may be that TMAO increases PD-induced inflammatory cell infiltration and the production of peritoneal inflammatory cytokines. In addition, studies have shown that TMAO not only causes primary peritoneal mesothelial cell necrosis, but also increases the synthesis of pro-inflammatory cytokines such as CCL2-α, TNF-α, IL-6, and IL-1β (Zhang et al., 2022). However, the specific mechanisms that cause elevated inflammatory markers are still unclear.

The implications of TMAO for patients with ESRD are not limited to its direct influence on renal function. Elevated levels of TMAO have been associated with potential disruptions in the efficacy of dialysis treatments, impacting the elimination of various harmful substances (Hai et al., 2015; Kalim et al., 2018; Mair et al., 2018). Furthermore, TMAO has been correlated with changes in the composition of gut microbiota, adding another layer of complexity to the multifaceted health challenges faced by individuals with ESRD (Bao et al., 2022). Moreover, studies indicate that over 50% of maintenance HD patients with ESRD experience protein energy wasting (PEW), which is linked to poor clinical outcomes (Chan et al., 2012; Ikizler et al., 2013). MHD patients with higher serum TMAO levels had lower BMI, triglyceride levels, DPI, and PEW, while 34% of those with low TMAO levels did not (Hu et al., 2022). Future studies must identify effective methods to demonstrate the specific mechanisms of action of PEW and TMAO.

In conclusion, TMAO plays a crucial role in the management and prognosis of ESRD. Elevated TMAO levels in ESRD patients not only signify a more advanced disease status but also play a role in the emergence and advancement of potentially fatal complications. Ongoing research on TMAO and its impact on ESRD has the potential to guide the development of more precise and efficient treatment approaches for this susceptible patient group.

## 4 Diverse strategies for modulating plasma TMAO levels

TMAO, derived from dietary choline, lecithin, and carnitine, is linked to cardiovascular, kidney diseases, and metabolic disorders. High blood TMAO levels increase the risk of these conditions, prompting research into interventions. Here, we explores strategies to control plasma TMAO levels and their public health implications (**Table 1**).

**Table 1:** Methods and drugs for inhibiting TMAO production.

categorization	Methods/drugs	Rationalization
	High-fat ketogenic diet(LCHF)	
Dietary patterns	Methionine-restricted diet	
	Mediterranean diet	
	ZDY01、ZDY04 Bifidobacterium	
	Resveratrol	
	Geraniol	
Microbiotherapy	Ligustrum robustum (Rxob)	Intestinal microorganisms
	Allicin	
	Curcumin Rhubarb enema	
	Human umbilical cord mesenchymal stem cells	
	3,3-Dimethyl-1-butanol (DMB)	CutC enzyme
	Iodomethylcholine (IMC) Fluoromethylcholine (FMC)	CutC enzyme CutC enzyme
	Phenylcholine	CutC enzyme
Intermediate blocking substance	3,3-diindolylmethane (DIM) Indole-3-carbinol (I3C) Fenugreekine	FMO3 enzyme FMO3 enzyme FMO3 enzyme
	Polymethoxyflavones (PMFs) Chlorogenic acid	FMO3 enzyme FMO3 enzyme
	Berberine	Intestinal microorganisms
	Finasteride	FMO3 enzyme
	Rosuvastatin	Lipoproteins, cholesterol
	Linacloroptide	F4/80-positive macrophages
	Suyin Detoxification Granule (SDG)	Intestinal microorganisms
	Hypoglycemic soup	Intestinal microorganisms
Drug	Chinese Herbal Formula clearing the veins and expelling blood stasis	Intestinal microorganisms
	Naringenin, paeoniflorin, beta-ecdysterone, 18beta-glycyrrhetinic acid, bitter amygdalin, leucovorin, shiba hu Saponin A Perilla Frutescens L	FMO3 enzyme ASK1-JNK Phosphorylation
	DXR IV	intestinal microorganisms

### 4.1 Dietary interventions

Dietary modifications play a significant role in influencing TMAO levels, with consumption of foods high in choline and carnitine. Reducing the intake of red and white meat (Wang et al., 2019), introducing new dietary modalities such as a high-fat ketogenic diet (LCHF) (Ang et al., 2020), dietary methionine restriction (Lu et al., 2023) and the mediterranean diet (Estruch et al., 2018) have all been found to be effective in reducing TMAO level in recent studies. It suggesting that dietary shifts towards plant-centric eating patterns could be beneficial. Additionally, certain food components can influence TMAO levels.

### 4.2 Gut microbiota manipulation

The gut microbiota plays a pivotal role in converting dietary components into TMAO, suggesting that modulation of the gut microbiota could serve as a viable approach to regulating TMAO levels. Probiotics like Lactobacillus plantarum ZDY01 and ZDY04 have demonstrated TMAO-lowering effects (Qiu et al., 2018; Tang et al., 2021). Similarly, bifidobacterium could regulate mouse gut microbiota (Wang et al., 2022). In addition, the intake of small molecules such as resveratrol (Hsu et al., 2020), geraniol (Lin et al., 2022), ligustrum robustum (Rxob) (Liu et al., 2021), allicin (Panyod et al., 2022), and curcumin (Zhang et al., 2022), rhubarb enema (Ji et al., 2021), implantation of human umbilical cord mesenchymal stem cells (Li et al., 2021), and even the fecal microbiota transplantation (FMT) (Smits et al., 2018) maintains a strikingly similar efficacy. However, further rigorous research is necessary to confirm safety, efficacy, and applicability of these interventions.

### 4.3 Related intermediate inhibitors

In TMAO metabolism, CutC/D & FMO3 are crucial. CutC/D regulates choline anaerobiosis, while FMO3 converts TMA to TMAO. Inhibiting these enzymes could inhibit the production of TMAO. Choline analog DMB mitigates TMAO, reducing its plasma levels and plaque formation (Wang et al., 2015; Brunt et al., 2022). In addition, novel cholinergic inhibitors of TMA lyase include iodomethylcholine (IMC) and fluoromethylcholine (FMC) could inhibit the production of TMAO (Pathak et al., 2020; Benson et al., 2023; Tain et al., 2023). In addition, choline analogs such as phenylcholine can also reduce TMA production (Schugar et al., 2022). Other choline analogs and compounds like DIM, I3C, fenugreekine, PMFs, and chlorogenic acid can also inhibit FMO3, decreasing TMAO levels (Anwar et al., 2018; Chen et al., 2019; Iglesias-Carres et al., 2023; Lee et al., 2023).

### 4.4 Pharmacological approaches

Although many drugs have been reported to target TMAO production, but further studies and clinical trials are needed to confirm their safety and efficacy. Fecal tests from metformin-treated donor mice showed that recipient mice had increased Bifidobacterium bifidum and Akkermansia, and decreased CutC/D in their gut microbiota (Su et al., 2021). Finasteride treatment may alleviate symptoms by inhibiting liver Fmo3 (Wang et al., 2022). Rosuvastatin may be associated with inhibition of HDL-cholesterol versus LDL-cholesterol production (Xiong et al., 2022). Linacloroptide can reduce TMAO levels, and improve kidney inflammation and fibrosis, as well as cardiac fibrosis, and contribute to the amelioration of CKD (Nanto-Hara et al., 2020). Besides, Suyin Detoxification Granule (SDG) have been found to prevent the progression of CKD by regulating intestinal microbiota, reducing circulating TMAO levels, inhibiting increased renal tubular iron concentration, suppressing pro-fibrotic factor secretion, and mitigating TMAO-induced renal fibrosis (Ge et al., 2024). Moreover, an increasing body of research has demonstrated that some herbal medicines such as berberine (Li et al., 2021), naringenin, paeoniflorin, beta-ecdysterone, 18beta-glycyrrhetinic acid, bitter amygdalin, leucovorin, shiba hu saponin A (Yu et al., 2021), Perilla frutescens L (Yong et al., 2023), and a number of herbal formulas (Ji et al., 2020; Zhang et al., 2021) that ameliorate metabolic abnormalities can also reduce the production of TMAO.

Regulating plasma TMAO levels requires a holistic approach, including diet changes, gut microbiota adjustments and medications. As research progresses, more effective interventions will emerge. Healthcare providers should monitor TMAO levels to better assess kidney, cardiovascular, and metabolic health, potentially reducing risks and enhancing outcomes.

## 5 Conclusion

TMAO has garnered more and more attention in the field of nephrology due to its essential role in many kidney diseases. The processes of its production, metabolism, and detection indicate that TMAO may be a valuable indicator of kidney health. TMAO has been implicated in the pathogenesis of many kidney diseases through mechanisms involving oxidative stress, inflammation, and endothelial dysfunction. Consequently, strategies aimed at modulating TMAO production or metabolism may be the novel therapeutic avenues for the prevent and treatment of kidney diseases. In conclusion, TMAO plays an essential role in the progression of many kidney diseases, and targeting the production or metabolism of TMAO may be an effective means to treat various kidney diseases.
